# Supplementary material for: Predicting visual function by interpreting a neuronal wiring diagram
Source: Nature. 2024 Oct 2;634(8032):113–23. doi: 10.1038/s41586-024-07953-5 (PMC11446822; doi:10.1038/s41586-024-07953-5)
Supplement: Supplementary file 6 — CRF and ERF predictions for individual TmY4 and TmY9 cells. Analogous to Supplementary Data 3, but for TmY target types. Shown are the top four monosynaptic pathways, the strongest pathway passing through each of the top ten intermediary types (ranking from Extended Data Fig. 7), and the trisynaptic pathway Tm1–TmY–Dm3–TmY (see the section entitled Prediction of spatial normalization). [file 41586_2024_7953_MOESM6_ESM.zip › DataS4/TmY9q/720575940631575493.pdf]

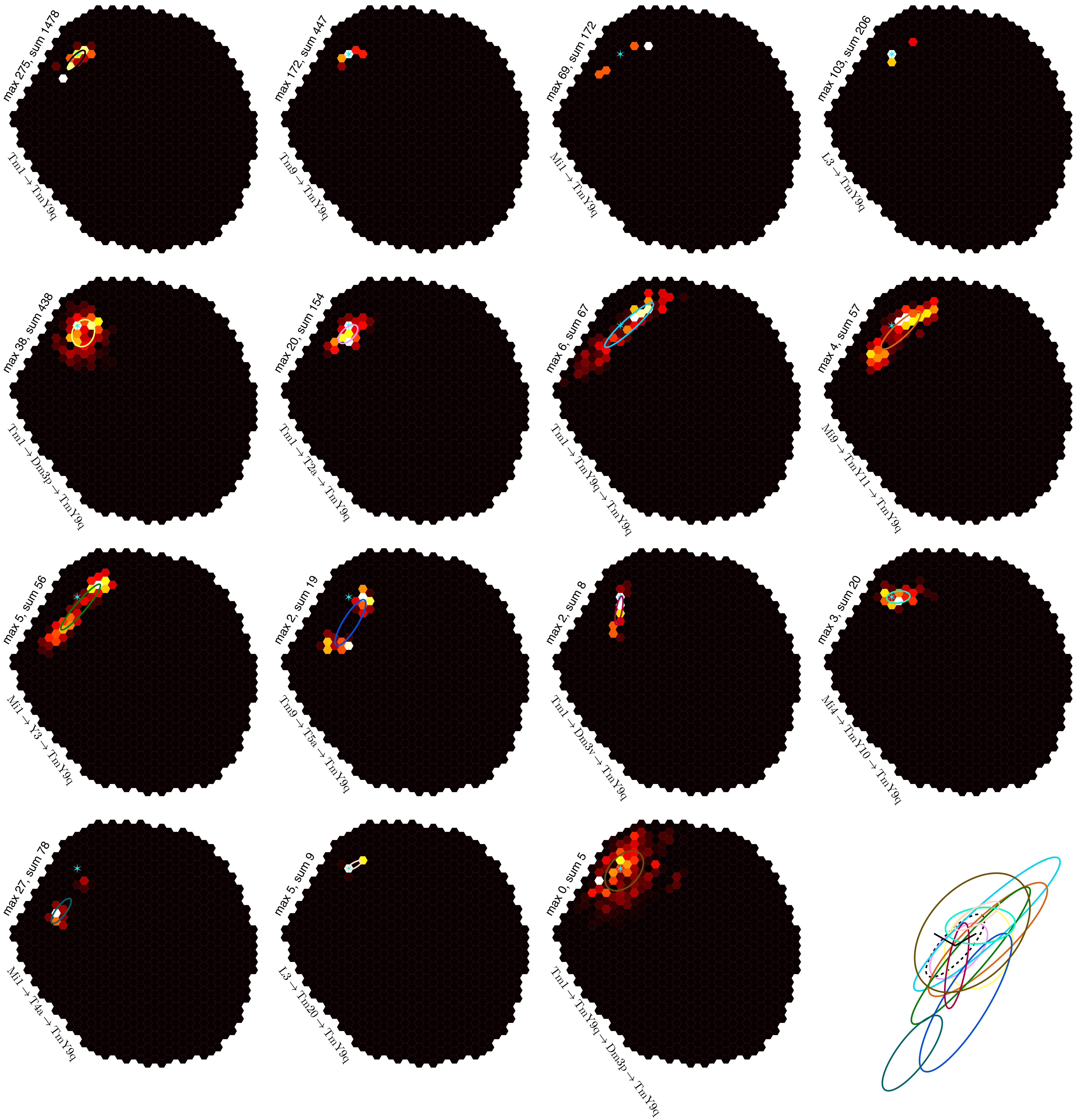

max 275, sum 1478
Tm1 → TmY9q
max 172, sum 447
Tm9 → TmY9q
max 69, sum 172
Mi1 → TmY9q
max 103, sum 206
L3 → TmY9q
max 38, sum 438
Tm1 → Dm3p → TmY9q
max 20, sum 154
Tm1 → T2a → TmY9q
max 6, sum 67
Tm1 → TmY9q → TmY9q
max 4, sum 57
Mi9 → TmY11 → TmY9q
max 5, sum 56
Mi1 → Y3 → TmY9q
max 2, sum 19
Tm9 → T5a → TmY9q
max 2, sum 8
Tm1 → Dm3v → TmY9q
max 3, sum 20
Mi4 → TmY10 → TmY9q
max 27, sum 78
Mi1 → T4a → TmY9q
max 5, sum 9
L3 → Tm20 → TmY9q
max 0, sum 5
Tm1 → TmY9q → Dm3p → TmY9q
